# Neurocompensatory Effects of the Default Network in Older Adults

**DOI:** 10.3389/fnagi.2019.00111

**Published:** 2019-06-04

**Authors:** Bryant M. Duda, Max M. Owens, Emily S. Hallowell, Lawrence H. Sweet

**Affiliations:** ^1^Department of Psychology, University of Georgia, Athens, GA, United States; ^2^Department of Psychiatry & Human Behavior, The Warren Alpert Medical School of Brown University, Providence, RI, United States

**Keywords:** older adults, neurocompensation, fMRI, default mode network, HAROLD

## Abstract

The hemispheric asymmetry reduction in older adults (HAROLD) is a neurocompensatory process that has been observed across several cognitive functions but has not yet been examined in relation to task-induced relative deactivations of the default mode network. The present study investigated the presence of HAROLD effects specific to neural activations and deactivations using a functional magnetic resonance imaging (fMRI) n-back paradigm. It was hypothesized that HAROLD effects would be identified in relative activations and deactivations during the paradigm, and that they would be associated with better 2-back performance. Forty-five older adults (*M* age = 63.8; range = 53–83) were administered a verbal n-back paradigm during fMRI. For each participant, the volume of brain response was summarized by left and right frontal regions of interest, and laterality indices (LI; i.e., left/right) were calculated to assess HAROLD effects. Group level results indicated that age was significantly and negatively correlated with LI (i.e., reduced left lateralization) for deactivations, but positively correlated with LI (i.e., increased left lateralization) for activations. The relationship between age and LI for deactivation was significantly moderated by performance level, revealing a stronger relationship between age and LI at higher levels of 2-back performance. Findings suggest that older adults may employ neurocompensatory processes specific to deactivations, and task-independent processes may be particularly sensitive to age-related neurocompensation.

## Introduction

The number of individuals aged 65 or older is projected to exceed 1.5 billion in 2050 (National Institute on Aging and World Health Organization, 2011) and will comprise approximately 30% of the population by 2060 (Parker et al., 2012). While this expansion is due in part to advancements in healthcare, the aging population will present new challenges for older adults (OAs) and their caretakers. In addition to OAs affected by neurodegenerative conditions, such as Alzheimer’s disease (Alzheimer’s Disease International, 2010), OAs without such conditions also exhibit well-studied patterns of neurocognitive decline. These include declines in processing speed (Salthouse, 1996; Charness, 2008), selective attention (Barr and Giambra, 1990; Madden, 1990), working memory (WM) (Balota et al., 2000; Zacks et al., 2000), and task-switching (Kray and Lindenberger, 2000; Kray et al., 2002). Underscoring their functional significance, these decrements have been linked to declines in independent living among healthy OAs (Suchy et al., 2011; Duda et al., 2014).

Consistent with cognitive findings, neuroimaging studies have identified patterns of neural changes in non-demented OAs. Structural neuroimaging studies have revealed age-related reductions in prefrontal and parietal white matter integrity (Persson et al., 2005; Grady, 2012) and hippocampal and prefrontal gray matter volumes (Haug and Eggers, 1991; Raz et al., 2004). Functional neuroimaging studies, using positron emission tomography (PET) and functional magnetic resonance (fMRI), have revealed age-related increases and decreases in task-related brain activity. These changes were initially thought to reflect poorer brain function underlying cognitive decline; decreased activation was thought to reflect a reduced allocation of neural resources, whereas increased activation was considered a marker of reduced efficiency and selectivity of neural responses, referred to as *dedifferentiation* (Li and Lindenberger, 1999; Li and Sikström, 2002; Grady, 2008). While dedifferentiation is still a viable explanation for some age-related neural changes, some patterns of alterations have been positively associated with cognitive function (Berlingeri et al., 2010) and have contributed to a general theory of *neural compensation* (Grady, 2012).

The theory of neurocompensation was initially invoked to explain observations of increased neural activity while OAs performed cognitive tasks as well as younger adults (YAs; Cabeza, 2002), or when increased activity positively correlated with performance only in OAs (McIntosh et al., 1999; Reuter-Lorenz et al., 2000). This view suggests that activation of alternate brain regions may serve to counteract cognitive decline (Cabeza, 2001, 2002; Cabeza et al., 2004). Evidence of age-related neurocompensation has been found using tests of episodic retrieval (Davis et al., 2008; McDonough et al., 2012), visuospatial skills (Lee et al., 2011), WM (Rypma et al., 2007; Carp et al., 2010), response inhibition (Hsieh and Fang, 2012; Sebastian et al., 2013), and selective attention skills (Davis et al., 2011; Ansado et al., 2012). These effects are frequently reported in the prefrontal cortex (PFC; Goh, 2011), which is consistent with prior literature of neurocompensatory processes in OAs (Cabeza et al., 1997; Madden et al., 1999a,b). These findings collectively support the age-related compensatory hypothesis, although several questions remain unanswered. For example, it is unclear which cognitive processes are supported by neurocompensatory mechanisms (Rajah and D’Esposito, 2005; Greenwood, 2007; Zarahn et al., 2007).

One prominent model of age-related neurocompensation is the hemispheric asymmetry reduction in OAs (HAROLD), which postulates that under similar circumstances, PFC activation during cognitive processes tends to be less lateralized in OAs than YAs (Cabeza, 2002). HAROLD and other age-related neurocompensatory theories offer different predictions of age-related neurocompensatory processes. For example, the compensation-related utilization of neural circuits hypothesis (CRUNCH) theory posits that the magnitude of network activation increases with task demand (Reuter-Lorenz and Cappell, 2008), while the scaffolding theory of aging and cognition (STAC) suggests a recruitment of additional networks when the primary network becomes inefficient (Park and Reuter-Lorenz, 2009). The HAROLD model is particularly attractive for studying age-related neurocompensation due to its focus on the PFC, which is consistent with the majority of age-related neurocompensatory findings (see Goh, 2011 for review). Moreover, patterns of activation consistent with HAROLD have been well-validated using a range of neuroimaging paradigms, from simple motor tasks (Mattay et al., 2002) to more complex tasks, such as verbal working memory (VWM) and episodic retrieval (Bäckman et al., 1997; Cabeza et al., 1997; Reuter-Lorenz et al., 2000; Morcom and Friston, 2012). HAROLD thus offers a useful framework for investigating age-related patterns of neurocompensation.

While the HAROLD model has been well-documented, several aspects of this model are not fully delineated. For example, some evidence suggests that HAROLD patterns observed in prefrontal regions may be specific to higher levels of task demand (Berlingeri et al., 2013). In addition, studies assessing the HAROLD activation pattern have not consistently evaluated or found support for the relation between neurocompensatory activity and behavioral performance. For example, using PET to investigate age-related neural changes in verbal and spatial WM, OAs showed an increased bilateral response in the PFC that was associated with slower reaction time but equivalent WM accuracy. There has also been some evidence to suggest the presence of HAROLD relative to functional connectivity (FC), though with limited evidence of cognitive change (Li et al., 2009).

Another aspect of the HAROLD model that has not been clarified is the distinction between relative activation and deactivation. Despite substantial evidence for HAROLD effects in task-related activation patterns, neurocompensation relative to task-independent neural processes have been understudied. The default mode network (DMN) has been defined by coactivation within a distributed network of cortical regions, which characterizes the resting state of the human brain (Greicius et al., 2004). Evidence suggests that several properties of the DMN are sensitive to the aging process (Spreng et al., 2016; Klaassens et al., 2017). First, reduced DMN coherence has been associated with declines in processing speed (Ng et al., 2016), WM (Hampson et al., 2006), and cognitive control (Turner and Spreng, 2015). Second, suppression of DMN regions (relative deactivation), which occurs during task engagement (Anticevic et al., 2012; Binder, 2012) is a marker of efficient executive functioning (EF) that becomes compromised in OAs (Duverne et al., 2008; Petrella et al., 2011; Spreng et al., 2016; Avelar-Pereira et al., 2017).

Evidence suggests that DMN deactivations may be particularly susceptible to aging and a potential contributor to neurocompensatory processes. For example, using fMRI and spatial judgment tasks, Park et al. (2010) reported significantly reduced deactivations in OAs, and importantly, faster reaction times were demonstrated by OAs who did effectively deactivate DMN regions. In addition, Miller et al. (2008) reported enhanced DMN deactivation in YAs that correlated with successful memory encoding; however, OAs did not show this pattern, and reduced deactivation was most evident among poorer performers. Using a verb generation task, Persson et al. (2007) also reported an age-related reduction in DMN deactivations that was associated with slower reaction time performance. Perhaps the strongest evidence of DMN-related neurocompensation is described by Davis et al. (2008), who developed the posterior-anterior shift in aging (PASA) hypothesis to explain the observation that OAs showed a pattern of reduced deactivations of posterior midline cortex, with increased deactivations of medial frontal cortex during a verb generation task. When matching groups on performance, OAs showed greater DMN deactivation of the left anterior cingulate and right anterior insula, as well as increased activations in the left middle frontal gyrus and right supramarginal gyrus. Both deactivation and activation effects were seen bilaterally, which, in the context of the relation between the DMN and task-positive network functions, suggests that the deactivations may also support cross-hemispheric neurocompensatory processes. Importantly, given the inverse correlation between DMN and task-positive networks that supports cognitive functioning (Fox et al., 2005), these bilateral effects suggest that an age-related neurocompensatory process (i.e., HAROLD) of relative deactivations may support successful task-positive functioning.

Further investigations of task-independent processes are needed to determine how the DMN and task-independent networks may interact with other models of neurocompensation, such as the HAROLD pattern in OAs. Since HAROLD and PASA models do not have direct support from studies of relative deactivation and given a lack of clarity in prior literature whether effects included summation of activation and relative deactivation or only activation effects, we considered examination of activation and relative deactivation separately to have high theoretical and methodological importance. The present study provides the first examination of HAROLD lateralization effects in both brain activations and relative deactivations in healthy OAs. Using fMRI during a WM paradigm, we evaluated the presence of the HAROLD pattern in the task-dependent brain response because neural activation associated with VWM has previously demonstrated sensitivity to age-related neurocompensatory processes, including the HAROLD effects. Second, using a resting fixation baseline to isolate suppression of baseline DMN activity, we evaluated the presence of HAROLD among task-induced deactivations. Moderation analyses were conducted to address whether observed HAROLD effects were associated with cognitive performance (i.e., n-back accuracy). Two homologous frontal lobe regions of interest (ROI) were used to calculate the laterality indices (LI) and examine the expected HAROLD effects. It was hypothesized that (1) OAs would demonstrate HAROLD patterns of task-elicited relative brain activation and deactivations, and (2) effects would be significantly moderated by 2-back performance, as a reflection of successful compensation.

## Materials and Methods

### Participants

Participants included 45 healthy, right handed English-speaking men and women over the age of 50 (25 women, age range 53–83, *M* age = 63.78 years, *SD* = 7.99) who comprised a healthy control group in a larger study of neurocognitive function in cardiovascular disease (Haley et al., 2009). These participants were recruited from the community via advertisements in the Providence, RI area. All participants underwent standard informed and written consent procedures. Assessments were conducted over three visits that spanned approximately 6 weeks and included a neuropsychological assessment, an echocardiogram, and an MRI scanning session. Exclusion criteria included left-hand dominance, corrected visual acuity poorer than 20:40, below 60% performance accuracy on the 2-back VWM task (i.e., < 1 *SD* from 50%), low global cognitive function (>1.5 *SD*s below the sample population on the Mini Mental Status Examination), or any MRI contraindications (e.g., metal implants). Significant medical (e.g., surgery, heart infarct), neurological (e.g., multiple sclerosis, traumatic brain injury with loss of consciousness), and psychiatric problems (e.g., substance abuse with hospitalization, diagnosis of any current psychiatric illness) were exclusion criteria that were assessed by interview, physical examination, review of medical records and self-report questionnaires. Participants were compensated for their participation. The study was approved and monitored by the university and hospital institutional review boards (IRB) where the research took place and conformed to the Helsinki Declaration on human subjects’ protection.

Demographic characteristics, estimated intellectual functioning, and 2-back performance are displayed in Table 1. The study sample comprised OAs with right-handed dominance (*M* = 86.7, *SD* = 14.40; range = 55–100; on a scale of 0-100, with increasing values representative of increasing right-dominance) and above average intellectual functioning (WTAR range = 96–119, mean = 111.53, *SD* = 6.40) and years of educational attainment (range = 12–21 years; mean = 16.36; *SD* = 1.96). WM performance (i.e., 2-back accuracy) was consistent with prior 2-back literature (Braver et al., 1997; Smith and Jonides, 1999; Sweet et al., 2008).

**Table 1:** Demographic and mean summary data.

Variable	Mean	SD	Min	Max
Age (years)	63.78	7.99	53.00	83.00
MMSE	29.40	0.70	28.00	30.00
Handedness (%R)	86.7	14.40	55.00	100.00
Education (years)	16.36	1.96	12.00	21.00
Predicted FSIQ	111.5	6.40	96.00	119.00
2-back accuracy	82.98	9.70	64.00	99.00

*MMSE, Mini mental status exam; Handedness was measured on a scale of 1-100, with increasing values representative of right-handed dominance, and all subjects reported right-handedness; Education, years of formal education attained; FSIQ, Full-scale intelligence quotient from the Wechsler Test of Adult Reading; 2-back accuracy includes correct hits and rejections proportional to the number of trials.*

### Behavioral Measures

**Verbal working memory paradigm.** The n-back paradigm was employed to challenge VWM systems during two imaging runs. The n-back has been widely used in functional neuroimaging research for more than 20 years and has the advantage of a well-described fMRI neural response (e.g., Braver et al., 1997; Smith and Jonides, 1999; Owen et al., 2005; Sweet et al., 2008). During the 2-back, six series of 15 consonants were presented visually for 500 ms each, with an interstimulus interval (ISI) of 2500 ms. Participants were asked to make a *yes* or *no* button-press response following each consonant to report whether or not it was the same as the consonant (irrespective of capitalization) presented two earlier in the series (e.g., underlined letters in the following sequence would be answered “yes”: w, N, r, N, R, Q, r, q, N, W, n …). Six 0-Back control blocks of nine consonants each were presented with the same duration of letter presentation and ISI. 0-Back blocks preceded each 2-Back block during the first imaging run and followed the 2-Back during the second run. Participants responded *yes* when a predetermined target consonant (“H” or “h”) appeared and *no* for other consonants. Consonant blocks of both conditions contained 33 percent “yes” targets in random locations within each series. Capitalization was randomized throughout to encourage verbal encoding. Two 27-s (27000 ms) blocks of resting fixation blocks were presented between the 0-Back/2-Back cycles. 2-back performance was calculated for each participant using the following formula: (number of correct positives + correct negatives)/ total number of letters presented. Two subjects were excluded from behavioral analyses due to near-chance 2-back performance (<60% accuracy). A diagram of the n-back task is presented in Supplementary Materials.

### Neuroimaging Measures

**MRI Acquisition.** Whole-brain echo-planar fMRI was conducted using a Siemens TIM Trio 3 tesla scanner (*TR* = 2500 ms, *TE* = 28 ms, *FOV* = 192^2^, matrix size = 64^2^, in 42 3-mm-thick axial slices). This procedure yielded 116 whole-brain volumes for each of the two 288s imaging runs, yielding a spatial resolution of 3 mm^3^ per voxel. Whole-brain high-resolution T1 images were also acquired in the sagittal plane for anatomical reference (*TR* = 1900 ms, *TE* = 2.98 ms, *FOV* = 256^2^ mm, matrix size 256^2^). Two conditions of the n-back paradigm (i.e., 2-back and 0-back) and a resting state “+” were presented using E-prime (Psychology Software Tools, Sharpsburg, MD, United States) and back-projected onto a screen visible to the participant via a mirror mounted to the head coil.

**MRI analysis.** MRI dataset processing and statistical analyses were performed with Analysis of Functional NeuroImages version 18.0.05 software (AFNI; Cox, 1996). Preprocessing of the functional runs included slice-time correction and registration of each volume to the third volume of the first imaging run to correct for head movement. Data from participants with head movement of > 3.0 mm in any direction (i.e., x, y, z, yaw, pitch, and roll) or movement greater than 0.3 mm on more than 25% of repetitions were omitted from analyses. The functional volumes were aligned to the anatomical volume in Talairach space. A 5-mm full-width half maximum Gaussian filter was applied and the raw time-series was scaled to a mean of 100. For each subject, the general linear model (GLM) was used to quantify 2-back activity relative to a resting state (i.e., fixation across) after controlling for 0-back active control task and other covariates (i.e., movement parameters) in order to facilitate examination of task-independent deactivations.

**Individual activity maps.** The resulting individual activity maps were thresholded (two-tailed α = 0.01) and corrected for multiple comparisons using AFNI’s false discovery rate (FDR; *q* = 0.05) procedure in order to provide a measure of volumetric activity, measured by significantly activated voxels beyond the threshold per ROI, for each participant.

**Group level processing.** For group level-analyses, two frontal ROIs were defined based on left or right hemisphere (i.e., relative to Talairach coordinate *x*-plane = 0) which extended to *y*-plane = 0. LIs, defined as left relative to right (left/right) frontal ROI response to the 2-back were calculated using each individual’s volume of significant response in each ROI (i.e., voxels of significant activity beyond threshold). Consistent with prior literature (Deblaere et al., 2004; Baciu et al., 2005; Yuan et al., 2006), an LI was calculated by subtracting intensity effects within the right frontal ROI from intensity effects within the left frontal ROI, then dividing this value by the average of mean activity across the two frontal ROIs (i.e., (left frontal - right frontal) / (left + right frontal). Thus, the scaling of LI was such that positive values indicate left-lateralized function and negative values indicate right-lateralized function. This process was conducted separately for relative activation and deactivation and repeated for volumes of significant activation and deactivation.

**Group level analyses.** Qualitative procedures were first conducted to examine the validity of brain activation patterns exhibited by the sample. In order to generate relative group level activation and deactivation maps for comparison to prior literature, the whole-brain unthresholded voxel-wise effects were tested against a hypothetical mean of zero (i.e., no 2-back effect) using one-sample *t*-tests. These whole-brain analyses were conducted using an FDR-corrected threshold of *p* = 10^-9^ (voxels greater than *t* = 8.42) with and a minimum of 10 contiguous voxels. Clusters of significant n-back response exhibited by our sample was compared to prior n-back literature (e.g., Smith and Jonides, 1999; Owen et al., 2005); in addition, the Neurosynth Image Decoder was used to quantify the concordance between the whole-brain results and neuroimaging studies included in the publicly available database. Next, hemispheric lateralization was examined. Consistent with prior literature, hemispheric dominance was determined by the size of the LI (Deblaere et al., 2004; Baciu et al., 2005; Yuan et al., 2006), following the criteria adopted by Yuan et al., 2006, in which an LI threshold of 0.10 (representative of 10 percent greater left relative to right hemispheric activity) was considered evidence of lateralization. Lastly, support for HAROLD effects were assessed by performing bivariate correlation analyses between age and each LI.

**Behavioral analyses.** Hierarchical multiple regression and moderation analyses were conducted using the Statistical Package for Social Sciences IBM SPSS Statistics 21.0 to test the influence of 2-back accuracy on HAROLD effects. Before conducting moderation analyses, assumptions of multiple linear regressions were examined, including homoscedasticity, independence of residuals and normality of residuals (Cohen et al., 2003). Multicollinearity between the independent variable and moderator were reduced through the use of centering (Aiken and West, 1991). For moderation analyses, the PROCESS SPSS macro plug-in (Hayes, 2012) was applied to examine the moderating influence of n-back performance on the relation between age and LI. For the present study, the interactions were visually probed in order to examine the nature of any moderation effects by examining conditional effects (i.e. simple slopes) at low (-1 *SD* below the mean) and high (+1 *SD* above the mean) levels of 2-back performance.

## Results

### Whole Brain Voxelwise Analyses

**FIGURE 1:**
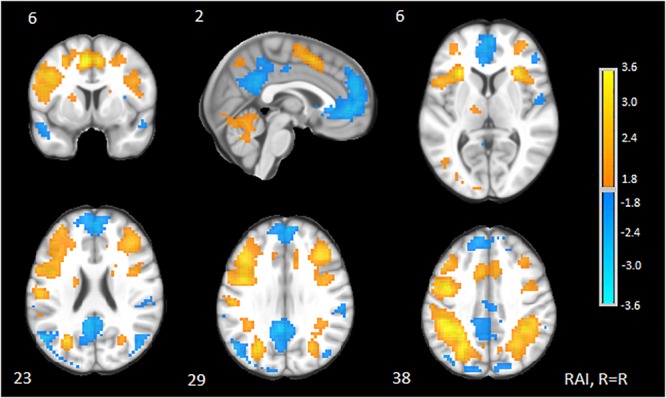
Clusters of significant neural response to 2-back. Representative of 2-back task-based activations and deactivations relative to a resting state baseline (“+”) from GLM analysis. Slices are provided in Talairach space and shown in RAI orientation (right = right). Yellow, activations; Blue, deactivations. Cluster centers of mass are reported in Table 2. Slice number (Z) is located above each slice. Color bars indicate magnitude effects for activations and deactivations.

The 2-back versus baseline contrast revealed widespread activation patterns commonly associated with n-back performance (Smith and Jonides, 1999; Owen et al., 2005), including the bilateral middle frontal gyrus, medial frontal gyrus, inferior parietal cortices, insula, and cerebellum. Relative deactivations were also consistent with prior literature (Anticevic et al., 2012; Binder, 2012) and overlapped substantially with regions associated with the DMN (Buckner et al., 2008), including the medial frontal gyrus/anterior cingulate, posterior cingulate, and superior frontal gyrus. Results from the Neurosynth Decoder, which was used to quantify the concordance of the present results to those in the publicly available database, indicated a value of 0.32 that was most consistent with task-specific measures of WM. See Figure 1 and Table 2 for neural patterns and related activity maps.

**Table 2:** Clusters of significant neural response to 2-back versus resting state.

Region	Voxels	x	y	z
L inferior parietal lobule	239	34	50	41
B posterior cingulate	231	0	–47	10
R cerebellum	151	–26	53	–22
L medial frontal gyrus	57	5	–5	51
L precentral/postcentral gyrus	51	35	22	53
B medial frontal	46	2	48	30
L inferior frontal/precentral gyrus	45	42	–3	33
L insula	37	29	–22	9
R inferior parietal lobule	36	–42	44	41
R insula	34	–31	–23	6
R medial frontal gyrus	25	–9	–15	45
L cerebellum	21	32	50	–26
R middle frontal gyrus	21	–39	–29	29
R superior parietal lobule	20	–30	60	42
L middle frontal gyrus	14	25	6	50

*L, Left; R, Right; B, Bilateral. Coordinates reported in center of mass Talairach space, RAI orientation. These clusters are shown in Figure 1.*

### Laterality and HAROLD Effects

Volumetric effects of the 2-back were summarized by the two frontal ROIs for both activations and deactivations. LIs were then calculated from these values and averaged at the group level. The *activation LI* yielded left-lateralization of this VWM task (*M* = 0.15, *SD* = 0.19) as expected (Jonides et al., 1997). Similarly, the relative *deactivation LI* was left-lateralized (*M* = 0.17, *SD* = 0.19). Descriptive statistics and the computed LI values are presented in Table 3.

**Table 3:** Neural effects of relative activations, deactivations, and laterality indices (LIs).

Regions	Mean	SD	LI
**Activations**		
Left Frontal	606	395	0.12^∗^
Right Frontal	478	324	
**Deactivations**		
Left Frontal	511	310	0.17^∗^
Right Frontal	363	233	

*ROIs, Regions of interest relative to task-induced activations and deactivations. Mean, Mean number of significantly active voxels within each frontal ROI; SD, Standard deviation of significantly active voxels within each frontal ROI. ^∗^Denotes left-lateralization of LI (Yuan et al., 2006).*

**FIGURE 2:**
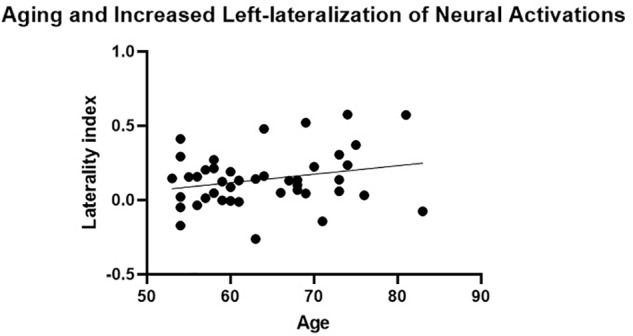
Aging and increased left-lateralization of neural activations. A significant and positive relationship between age and laterality index of neural activations was not considered consistent with the neural pattern of the hemispheric asymmetry reduction in older adults (HAROLD) effect.

**FIGURE 3:**
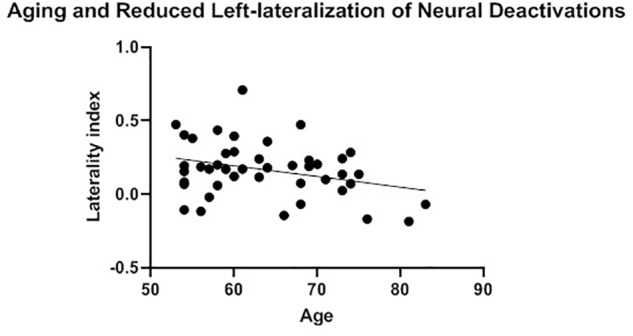
Aging and reduced left-lateralization of neural deactivations. A significant and positive relationship between age and laterality index of neural deactivations was considered consistent with the neural pattern of the hemispheric asymmetry reduction in older adults (HAROLD) effect.

The presence of HAROLD was then examined via bivariate Pearson correlations between age and each LI. The LI of neural activations was significantly and positively associated with age for volumetric effects *r*(42) = 0.34, *p* = 0.02 (see Table 4). Consistent with the HAROLD model predictions, the LI of neural deactivations were significantly and negatively associated with age *r*(42) = -0.31, *p* = 0.04 (see Table 4). Scatter plots of the relation between age and LIs for relative activations and deactivations are presented in Figure 2, 3, respectively, with residuals shown in Supplementary Materials.

**Table 4:** Correlations among demographics, cognition, and LI.

Variable	Age	Education	n-back
**Activations**		
Age			
Education	–0.02		
n-back	–0.18	0.05	
Laterality Index	0.34ˆ*	0.08	–0.15
**Deactivations**		
Age			
Education	–0.02		
n-back	–0.18	0.04	
Laterality Index	–0.31ˆ*	0.14	0.37ˆ*

*^∗^*p* < 0.05. A significant relation between age and the laterality index of neural deactivations was interpreted as consistent with the HAROLD effects.*

### Moderating Effect of 2-Back Accuracy

Hierarchical multiple regression and moderation analyses were conducted to examine the influence of 2-back performance on the relation between age and LIs. Two models were used to explore the four statistically significant relations between age and LIs of relative activations and deactivations.

For neural activations, age significantly predicted LI (*b* = 0.01, *p* = 0.03). However, 2-back accuracy (*b* = 0.00, *p* = 0.98), and the age × 2-back accuracy interaction term (*b* = 0.00, *p* = 0.96) were not significantly predictive of LI (total *R*^2^ = 0.13, *p* = 0.96). Moderation analysis specific to neural deactivations, however, yielded significant effects. Over and above age (*b* = -0.01; *p* = 0.02), 2-back accuracy (*b* = 0.01; *p* = 0.05) was a significant predictor of unique variance in LI (total *R*^2^ = 0.28; *F*(3, 40) = 5.09, *p* < 0.001). In addition, the interaction term of 2-back accuracy x age was a significant predictor of unique variance in LI over and above age and 2-back accuracy (Δ*R*^2^ = 0.08; Δ*F*(3,40) = 4.33; *b* = 0.00; *p* = 0.04). Results of this moderation effect are presented in Table 5.

**Table 5:** 2-back accuracy moderates age and deactivation laterality.

Total Model	R^2^	*SE*	F	*p*	ΔR^2^	
	0.28	0.03	5.09	0.00	0.08	
			
Model	*b*	*SE*	t	*p*	LLCI	ULCI
**Main Effects**		
Age	–0.01	0.00	–2.33	0.02	–0.01	0.00
n-back	0.01	0.00	2.02	0.05	0.00	0.01
**Interaction**		
Age ^∗^ n-back	0.00	0.00	–2.08	0.04	0.00	0.00

*^∗^*p* < 0.05.*

**FIGURE 4:**
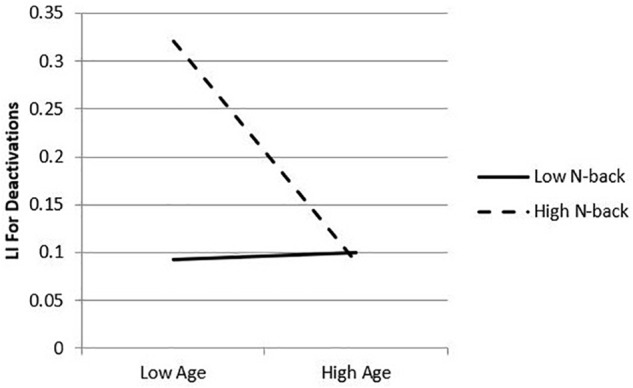
Simple slopes plot of conditional effects of 2-back. 2-back performance moderates the relation between age and laterality index. Plot represents laterality index (greater number depicts greater left lateralization) as predicted by age at fixed values of the moderator, 2-back: +1 standard deviation (11.5) and –1 standard deviation (–11.5) from the sample mean.

Given evidence of an interaction effect between age and 2-back accuracy on the relative deactivation LI, simple slopes analyses were conducted by estimating the conditional effect of age at specific values of 2-back performance (in this case, ± 1 SD from the sample mean, or -11.50 and 11.50, respectively) and tested whether the slopes were statistically significant from zero by a null hypothesis test (see Hayes, 2013). Results of these analyses revealed an association between age and LI, with age significantly related to LI for higher levels of 2-back performance (*b* = -0.01, *p* = 0.01, 95% *CI*s = -0.05, -0.00) but not significantly related to LI for lower levels (*b* = -0.00, *p* = 0.82, 95% *CI*s = -0.01, 0.01). In other words, high 2-back performers exhibited a stronger negative relationship between age and deactivation LI, or HAROLD effects. A plot of the simple slopes analyses is presented in Figure 4.

## Discussion

Hemispheric asymmetry reduction in older adults is a well-established neurocompensatory process that has been consistently demonstrated to support healthy OAs maintenance of cognitive function (Reuter-Lorenz et al., 2000; Cabeza, 2002; Morcom and Friston, 2012). However, to date, no studies have investigated HAROLD effects on relative deactivations of the DMN. The present study sought to determine if (a) HAROLD would generalize to task-induced activations and deactivations using a verbal n-back paradigm, and (b) if HAROLD effects would be significantly and negatively associated with 2-back performance. Results of whole-brain voxelwise analyses revealed expected patterns of neural response associated with the 2-back vs. a resting state baseline (Owen et al., 2005), including deactivations in DMN regions (Buckner et al., 2008). LIs calculated from 2-back-associated neural activation indicated left-lateralization that was consistent with previous findings (Smith and Jonides, 1999; Owen et al., 2005). Extending this finding, the deactivation LI also indicated left-lateralization. Regarding neurocompensatory processes, OAs demonstrated an unexpected age-related increase in left-lateralized activity that was not overtly associated with 2-back performance. In contrast, and consistent with our second hypothesis, an age-related change in the deactivation LI reflected a HAROLD pattern that was influenced by 2-back performance, such that high- but not low-performers demonstrated this shift.

The significant positive correlation between age and activation LI suggests that an age-related increase in left hemispheric brain response may have supported OAs’ maintenance of cognitive functioning. While this finding appears to contradict the HAROLD model, it is consistent with evidence supporting the presence of other neurocognitive compensatory processes in OAs. For example, differences in capacity (i.e., the degree to which a brain network is maximally recruited to perform a task) has been associated with cognitive maintenance (Bosch et al., 2010). At the network level, the compensation-related utilization of neural circuits hypothesis (CRUNCH) theory predicts that increased cognitive demands will result in a concomitant increase in network function (Reuter-Lorenz and Cappell, 2008). In the same vein, evidence has supported the STAC, which posits that recruitment of additional neural circuits or even networks occur when the primary networks have become inefficient or damaged due to pathology (Park and Reuter-Lorenz, 2009). Thus, one or more broader theories of compensation may complement the HAROLD model as it relates to successful maintenance of VWM. The younger age range of the present sample, relative to the age of participants in prior HAROLD studies (Cabeza, 2002) is not inconsistent with more traditional compensatory models.

Uncertainty regarding the function of overactivation, however, has led to increased interest in cognitive performance correlates. For example, overactivation among OAs has been linked to both reduced (Logan et al., 2002; Milham et al., 2002; Johnson et al., 2004; Park et al., 2004; Thomsen et al., 2004) and maintained cognitive performance (Grady et al., 2003; Persson et al., 2004; Rossi et al., 2004; Gutchess et al., 2005) which have been interpreted to reflect dedifferentiation and compensation, respectively. In the present study, 2-back accuracy did not moderate the relation between age and LI for activations, suggesting that increased left-lateralized activity did not function to maintain performance. However, lack of an observable relation between overactivation and performance may not necessarily mean that these alterations are not compensatory in nature; for example, compensatory increases in bilateral recruitment may not be extensive enough to offset opposite-hemisphere reduction in efficiency (i.e., resulting in performance declines), or individual differences may not be related to performance on a specific task (see Grady, 2012 for review). Importantly, while higher performing OAs in the current study did not evidence a greater degree of overactivation, lower performing OAs also did not. It is therefore plausible that the lower performing OAs maintained previous levels of cognitive function prior to age-related decline via increased left-lateralized activation.

Findings of the present study support the notion that relative deactivations are sensitive to the aging process, lateralized similarly to task-induced activations, and associated with successful task performance on a measure of VMW. A significant negative correlation between age and the deactivation LI, coupled with maintained 2-back performance (i.e., > 60% for each participant) suggests that reduced asymmetry of deactivations supported the OAs’ maintenance of cognitive functioning. Therefore, the current results provide evidence of a novel HAROLD finding, extending previous findings to deactivations, and more specifically, via a within-group fMRI study of community-dwelling OAs. This finding is further strengthened by an observed moderation effect of 2-back accuracy, suggesting that reduced asymmetry of deactivations may support maintenance of baseline cognitive abilities. Results of simple slopes analyses, conducted to elucidate the nature of this effect, revealed that higher performing OAs demonstrated an increased reduction of asymmetry specific to deactivations, while lower performing OAs did not (see Figure 2). This is consistent with the growing expectation that neurocompensatory processes relate to an observable maintenance in cognition.

Due in part to observations of their importance in neurocognitive function, task-induced deactivations of the DMN have become the target of increased investigation (Anticevic et al., 2012; Binder, 2012; Gilbert et al., 2012; Spreng, 2012; Hansen et al., 2014; Spreng et al., 2016). Evidence suggests that deactivations are reduced in the aging process (Lustig et al., 2003; Rombouts et al., 2005; Grady et al., 2006) and associated with poorer task performance (Persson et al., 2007). The present findings suggest that DMN deactivations may also help to explain neurocompensatory processes. This notion is further substantiated by the relation between DMN and task-positive network functioning (Anticevic et al., 2012; Spreng et al., 2016); for example, cross-hemispheric neurocompensatory activations (e.g., HAROLD) may correspond with cross-hemispheric alterations in DMN deactivation patterns that are required for effective reallocation of neural resources.

Consistent with this notion, the PASA model has been used to explain an age-related posterior-anterior shift of task-related DMN deactivations that appear to support cognitive function, such as semantic fluency (Davis et al., 2008). Others have since identified general age-related reductions in DMN deactivation that were associated with slower reaction time on measures of spatial skills and higher scores on tests of EF (Persson et al., 2007; Park et al., 2010). Results of the present study provide further evidence that DMN deactivations may be particularly important for the maintenance of cognitive function in the face of age-related decline and warrant future investigations of neurocompensatory processes.

### Limitations

Few studies have investigated HAROLD effects specific to WM among healthy OAs, and most were older PET studies. Prior literature has also focused almost exclusively on group differences (i.e., YA vs. OA) in HAROLD effects specific to task-dependent activations associated with WM. Interpretation of our findings by comparison to prior studies is therefore limited. A YA comparison group would have aided the interpretation of results. In addition, the sample of the present study was relatively young with respect to the aging literature (range = 53 to 83 years; mean = 63.78, *SD* = 7.99), as researchers often sample OA populations from 65 years and beyond. Interpretation of study findings are also limited by a relatively small sample size (i.e., not adequately powered to detect small effects). The sample was above average in intelligence, well-educated, and predominantly of Caucasian ethnicity (96%), which poses potential problems with generalizability.

### Future Directions

As the first investigation of age-related task-independent neurocompensatory processes utilizing a within-group experimental design, findings of the current study warrant replication. Consistent with the CRUNCH model and a prior study of HAROLD (Berlingeri et al., 2013), results of the present study suggest that multiple compensatory processes may support cognitive function in old age. Given our identification of dual neurocompensatory processes (i.e., bilateralization of task-independent deactivations and increasing left-lateralization of task-related activations) during a VWM paradigm, future investigations may also benefit from consideration of both task-dependent and task-independent brain responses in the context of newer models of neurocompensation such as CRUNCH and STAC. Evidence of HAROLD effects among relative to deactivations suggest that baseline DMN processing may be altered in OAs. Therefore, decline in cognitive performance and associated task-related brain response may be explained, in part, by changes in baseline processing.

A better understanding of factors that influence neurocompensatory processes may aide healthy OAs in maintaining cognitive function and *aging gracefully.* For example, several sociological and cultural factors have been linked to maintenance of cognitive function in aging, such as education, occupational complexity, social activity, and physical exercise (Katzman, 1993; Valenzuela et al., 2008; Stern, 2012), and brain changes associated with these factors is a topic of growing interest (Barulli and Stern, 2013; Stern, 2016). Future research of age-related neurocompensatory processes may also aide researchers and clinicians in identifying problem areas in OAs (e.g., less efficient processing, overactivations and performance declines), and those at-risk of developing neurodegenerative conditions. Early identification of at-risk OAs may in turn inform developing cognitive training programs, which refer to a range of structured programs (e.g., computerized tasks engaging EF systems) intended to maintain cognition or ameliorate cognitive deficits (Bahar-Fuchs et al., 2013). A growing literature suggests that cognitive (Karbach and Verhaeghen, 2014) and physical exercise interventions (Bherer, 2015) induce neural changes associated with improved cognitive performance (for review, see Bamidis et al., 2014) and appear to be a promising future complement to pharmacological interventions for OAs and warrants continued research.

## Ethics Statement

The study was approved and monitored by the university and hospital institutional review boards (IRB) where the research took place and conformed to the Helsinki Declaration on human subjects’ protection. All participants underwent standard informed and written consent procedures.

## Author Contributions

BD, MO, EH, and LS contributed to the design and implementation of the research, analysis of the results, and writing of the manuscript.

## Conflict of Interest Statement

The authors declare that the research was conducted in the absence of any commercial or financial relationships that could be construed as a potential conflict of interest.
