# Supplementary figures and images for: Neurocompensatory Effects of the Default Network in Older Adults
Source: Front Aging Neurosci. 2019 Jun 4;11:111. doi: 10.3389/fnagi.2019.00111 (PMC6558200; doi:10.3389/fnagi.2019.00111)

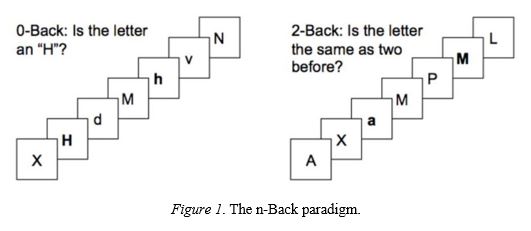

Supplement: Supplementary file 1 [file Image_1.JPEG]

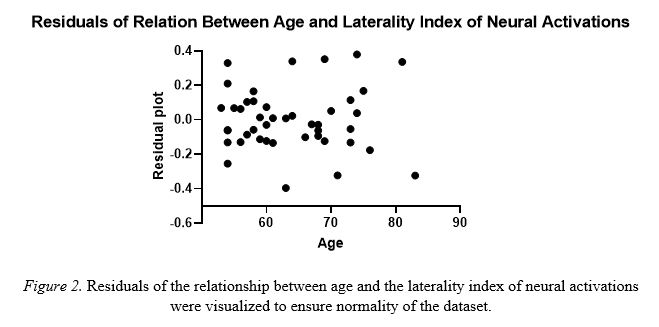

Supplement: Supplementary file 2 [file Image_2.JPEG]

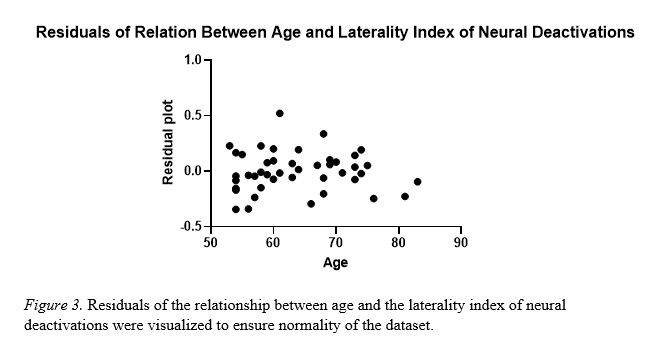

Supplement: Supplementary file 3 [file Image_3.JPEG]
